# Ethical Family Interventions for Childhood Obesity

**Published:** 2011-08-15

**Authors:** Mandy L. Perryman

**Affiliations:** Lynchburg College

No dialogue about ethical interventions in the treatment of childhood obesity would be complete without including the role of family, particularly parents, in influencing their child’s diet and physical activity. However, health experts have been hesitant to address this issue. Ethical concerns for family-based interventions include parents’ rights and responsibilities to protect their children, perceptions of obesity as child abuse or neglect, and the parents’ role as decision makers on their child’s behalf because of the child’s limited capacity to comprehend the risks and benefits of treatment. Family-based interventions are programs that target parents and children in creating a healthy lifestyle, which is difficult as families are confronted with an obesogenic food environment and have sedentary behaviors. Interventions that focus on improving overall family health are an ethical and effective way to decrease childhood obesity.

At a young age, children learn to assimilate their parents’ health-related beliefs and behaviors; therefore, environment and genetics can contribute to childhood obesity. In a family with 1 overweight parent, the child has a 40% chance of becoming overweight (1). If both parents are overweight, the risk increases to 80%, compared with 7% in a family in which neither parent is overweight (1). The overweight parent is considerably more likely to diet and make disparaging remarks about himself or herself in the presence of the obese child (2). These behaviors model an unhealthy self-concept for the child, which can result in an inferior body image and low self-esteem. Therefore, incorporating the family in ethical interventions for childhood obesity is imperative.

## Parents' Rights and Responsibilities

Within legal boundaries, parents have the right to raise their children as they wish, and they have the responsibility to protect their children from harm. This creates an ethical dilemma when children become obese. Because parents have the right to raise their children according to their own value system, the choices that parents make for themselves concerning diet and physical activity are likely to be the same choices that they make for their children. The decisions that parents make about the family's lifestyle affect their child's current and future mental and physical health. Since parents have the right to manage the nutrition and activity of their children, they are ultimately responsible for their child's obesity. Though childhood obesity is far more complex than parental choices alone, and no one decision or action can cause obesity, some child health advocates suggest that, by failing to prevent obesity, parents are accountable for indirect harm or negligence to their child (3).

## Child Abuse or Neglect

Legally, child abuse is often defined as behavior or lack of action that results in damage to a child or puts a child at risk of injury. Ethically, parents have an obligation to provide for their child's needs and to do no harm. Severe or chronic abuse or neglect can lead to the involuntary termination of parental rights and criminal charges. In 2008, the Child Welfare League of America reported that many state courts have expanded their definition of medical neglect to include morbid obesity and then ruled that certain children were victims of neglect because of their obesity (4). For example, the mother of a 14-year-old was arrested and charged with criminal neglect because her son weighed 555 pounds (5). Some child health advocates support such decisions and view childhood obesity as harm to the child by the parent.

## Parents as Decision Makers

Parents act as decision makers for their children in the areas of nutrition and activity because children do not yet possess the maturity and capacity needed to make health-related choices. This is an ethical issue because parents are acting on the child's behalf while having a vested interest in the outcome of those choices. Consequently, parents are biased to their own worldview and are inclined to make decisions for the child that benefit themselves, the family, or both. Parents make food choices, monitor sedentary behaviors, and engage children in physical and social activities.

Decisions made by parents of an obese child may include putting the child on a diet. This can be isolating for the child and may lead to further body dissatisfaction. One study reported that 50% of children in grades 3 to 6 wanted to be thinner, and approximately one-third of them were actively trying to change their weight (6). Seventy-five percent of these children reported having learned about dieting from someone in their family, usually a parent (6). In other studies, half of children aged 9 to 11 years were sometimes or very often on diets, and 82% of their families were sometimes or very often on diets (7). Continuing to put young children on restrictive diets perpetuates the cycle of weight loss–weight gain and reinforces a negative self-concept.

Parents may choose a more inclusive method to address childhood obesity: family-based interventions. Family-based interventions are community-based public health programs that empower the entire family to reduce sedentary behaviors (eg, watching television, playing video games, using the computer) and to increase good nutritional choices (eg, eating fresh fruits and vegetables). The family also practices problem solving and begins to restructure its thinking to change learned unhealthy behavior patterns (eg, snacking when stressed, cleaning one's plate). Family-based models have been implemented since the 1980s, and although their design and execution vary, familial involvement and positive support have been demonstrated to be important for reducing childhood obesity (8). The role of the parent in family-based interventions is to reinforce healthy behaviors, reward optimal behaviors without using food, set consistent meal and snack times, offer nutritious foods, remove unhealthy foods from the home, and model desired behaviors.

Family-based interventions addressing ethical concerns are possible, as illustrated by the National Institutes of Health's We Can! (Ways to Enhance Children's Activity and Nutrition) program (9). This program offers tools and resources for families, health care providers, and communities. Significant improvements in knowledge, attitude, and behaviors were measured in parents and children who participated in the program (9). We Can! provides strategies to eat well and be physically active for families of all economic backgrounds.

Barriers to family-based interventions include resource accessibility related to familial socioeconomic status, time caregivers can spend at home, and food availability within communities. For instance, health-focused programs for families, such as the walking school bus, might be offered only in certain areas. The walking school bus is an initiative in which parents walk groups of children to school, thus increasing physical activity (10). However, neighborhoods with dilapidated or no sidewalks, heavy traffic, or high crime rates might not be able to implement a walking school bus. Time and resources for shopping and cooking are also needed for parents to prepare nutritious meals and promote family activity. Multiple jobs and financial obligations make that difficult for certain families. Family-based interventions must be tailored to include parents with limited income. Requiring parents to provide resources they are unable to afford is an example of an unethical intervention. Lastly, substantial attention has been given to today's food deserts: urban areas inhabited by ethnically diverse families where nutritious food is scarce and expensive. Often when families do want to buy more healthful foods, these foods are not readily available to them.

## Conclusion

Family dynamics play a major role in childhood obesity; yet, health experts have been reluctant to acknowledge the family in ethical interventions for childhood obesity. As interventions are developed, consideration needs to be given to societal factors, such as the obesogenic food environment, the propensity toward sedentary behaviors, and the limited financial resources of communities. Family factors must also inform the conception of childhood obesity interventions, such as parents' rights and responsibilities to protect their children, perceptions of obesity as child abuse or neglect, and the parents' role as decision makers on their child's behalf. Through ethical family-based interventions focused on nutrition and physical activity, the entire family can create and maintain a healthy lifestyle, which is essential in preventing and treating childhood obesity.
